# Long-term outcomes of low-dose dasatinib in older patients with chronic myeloid leukemia in chronic phase: an extended follow-up of the DAVLEC phase 2 trial

**DOI:** 10.1038/s41408-026-01526-7

**Published:** 2026-05-28

**Authors:** Hiroshi Ureshino, Kazunori Murai, Takashi Kumagai, Tatsuji Mino, Kaichi Nishiwaki, Satoshi Wakita, Tomoharu Takeoka, Masayuki Mita, Chikashi Yoshida, Nobuhiko Uoshima, Toshihiro Fukushima, Junya Kuroda, Takeshi Kondo, Masahiro Chiba, Takuya Miyazaki, Katsuhiro Io, Tomoaki Ueda, Takaaki Ono, Kensuke Usuki, Takahiro Yamauchi, Katsumichi Fujimaki, Takayuki Ikezoe, Atsushi Kawaguchi, Shinya Kimura

**Affiliations:** 1 Division of Hematology, Respiratory Medicine and Oncology, Department of Internal Medicine, Faculty of Medicine, Saga University, Saga, Japan; 2 Department of Hematology, Iwate Prefectural Central Hospital, Morioka, Japan; 3 Department of Hematology, Ome Medical Center, Ome, Japan; 4 Department of Hematology, Hiroshima City Asa Hospital, Hiroshima, Japan; 5 Division of Clinical Oncology and Hematology, Department of Internal Medicine, The Jikei University Kashiwa Hospital, Kashiwa, Japan; 6 Department of Hematology, Nippon Medical School, Tokyo, Japan; 7 Division of Hematology and Immunology, Japanese Red Cross Otsu Hospital, Otsu, Japan; 8 Department of Hematology, Shirakawa Kosei General Hospital, Shirakawa, Japan; 9 Department of Hematology, National Hospital Organization Mito Medical Center, Ibaraki, Japan; 10 Department of Hematology, Japanese Red Cross Kyoto Daini Hospital, Kyoto, Japan; 11 Department of Hematology and Immunology, Kanazawa Medical University, Kanazawa, Japan; 12 Division of Hematology and Oncology, Department of Medicine, Kyoto Prefectural University of Medicine, Kyoto, Japan; 13 Blood Disorders Center, Aiiku Hospital, Sapporo, Japan; 14 Department of Hematology, Asahikawa City Hospital, Asahikawa, Japan; 15 Department of Hematology, Yokohama City University Medical Center, Yokohama, Japan; 16 Department of Hematology, Kansai Electric Power Hospital, Osaka, Japan; 17 Department of Hematology and Oncology, The University of Osaka, Suita, Japan; 18 Divison of Hematology, Hamamatsu University School of Medicine, Hamamatsu, Japan; 19 Forth Department of Internal Medicine, Mizonokuchi Hospital, Teikyo University School of Medicine, Itabashi City, Japan; 20 Department of Hematology and Oncology, Faculty of Medical Sciences, University of Fukui, Fukui, Japan; 21 Department of Hematology, Fujisawa City Hospital, Fujisawa, Japan; 22 Department of Hematology, Fukushima Medical University, Fukushima, Japan; 23 Education and Research Center for Community Medicine, Faculty of Medicine, Saga University, Saga, Japan

**Keywords:** Myeloproliferative disease, Phase II trials

## To the Editor

BCR::ABL1 tyrosine kinase inhibitors (TKIs) have significantly improved the survival outcomes of patients with chronic myeloid leukemia in chronic phase (CML-CP) [1]. Older patients account for a substantial proportion of the CML population, with >20% of the patients diagnosed at ≥70 years of age. This group frequently presents with multiple comorbidities and polypharmacy, which both increase their vulnerability to treatment-related adverse events (AEs) and complicate long-term TKI administration [2]. The DAsatinib, Very Low-dose, for Elderly CML-CP patients (DAVLEC) trial, which was a multicenter, single-arm, phase 2 study that we previously reported [3], prospectively evaluated the safety and efficacy of low-dose dasatinib (20 mg daily) as frontline therapy in newly diagnosed CML-CP patients aged ≥70 years. In the primary analysis, low-dose dasatinib achieved a 12-month major molecular response (MMR) rate that was non-inferior to historical benchmarks [4], with a favorable safety profile. Based on these findings, it is suggested that dose-optimized dasatinib might offer a favorable balance between efficacy and tolerability in older patients [5]. Given that CML is a lifelong disease, long-term follow-up is essential. Thus, in the present study, we aimed to evaluate the long-term efficacy and safety of low-dose dasatinib in an elderly population using extended follow-up data from the DAVLEC cohort.

The DAVLEC trial was conducted at 25 hospitals in Japan. Its original trial design and primary results have been reported previously [3]. Patients were treated according to the DAVLEC trial protocol. After completing the protocol-defined 12-month treatment period, patients who continued dasatinib therapy or not at the discretion of the treating physician were followed as part of a long-term observational follow-up. The endpoints of this long-term follow-up study were as follows: cumulative rates of MMR, MR^4.0^, and MR^4.5^; progression to the accelerated or blast phase; overall survival (OS); incidence of grade 3–4 AEs throughout the follow-up period; discontinuation of dasatinib therapy during follow-up; and exploration of prognostic factors. All statistical analyses were performed using EZR [6]. The detailed methods are presented in the Supplementary Methods. The study was conducted in accordance with the Declaration of Helsinki and was approved by the institutional review board of Saga University as well as the institutional review boards of all participating centers (approval number: **2024-07-04**). The original DAVLEC study was registered with the UMIN Clinical Trials Registry (**UMIN000024548**). All patients provided written informed consent for participation and long-term data collection.

Between October 1, 2024 and December 31, 2025, 48 patients with newly diagnosed CML-CP who had participated in the previous phase 2 DAVLEC trial were recruited from 22 centers in Japan. The patients’ median age at diagnosis was 78 years (interquartile range [IQR]: 74.8–83.5 years). At study entry, 12 (25.0%) of the 48 patients had cardiovascular disease, and 26 (54.2%) had cardiovascular risk factors. Two patients had chronic obstructive pulmonary disease, and one had asthma. Additionally, 17 (35.4%) patients had a history of other malignancies. Tables S1 and S2 show the detailed patient characteristics.

**Fig. 1:**
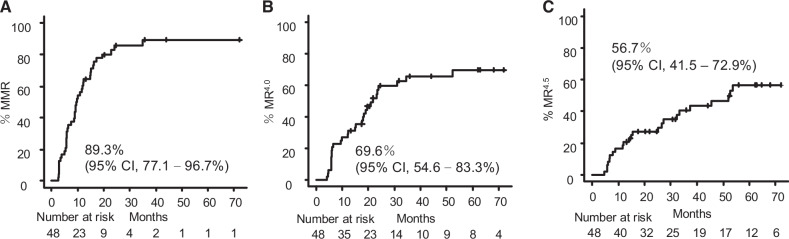
Cumulative molecular responses. The cumulative incidence of major molecular response (MMR) (**A**), MR^4.0^ (**B**), and MR^4.5^ (**C**). Numbers at risk are shown below each panel.

The 5-year cumulative incidence rates of MMR, MR^4.0^, and MR^4.5^ were 89.3% (95% confidence interval [CI]: 77.1–96.7%), 69.6% (95% CI: 54.6%–83.3%), and 56.7% (95% CI: 41.5–72.9%), respectively, with a median follow-up duration of 68.1 months (IQR, 38.2–76.0 months) (Fig. 1A-C). Notably, among the 48 patients, 26 (54.2%) achieved MMR at a dasatinib dose of ≤20 mg (Table S3).

Patients achieving an early molecular response (EMR) had a significantly higher likelihood of attaining MMR (hazard ratio [HR]: 2.556, 95% CI: 1.056–6.185, *p* = 0.037). At 12 months, 27 (56.3%) of the 48 patients achieved MMR, which was considered an optimal response. Patients achieving MMR at 12 months were significantly more likely to attain MR^4.0^ (HR: 6.196, 95% CI: 2.484–15.450, *p* < 0.001) and MR^4.5^ (HR: 5.044, 95% CI: 1.710–14.880, *p* = 0.003; Figure S1).

The proportions of most T- and NK-cell subsets increased significantly from baseline to 12 months (Table S4) [7]. Patients with a higher proportion of CD3^-^CD56^+^ or CD16⁺CD56⁺ NK cells at treatment initiation of dasatinib were more likely to achieve MMR (Tables S5 and S6). Univariate analysis showed that male sex was a significant unfavorable prognostic factor associated with a lower likelihood of achieving MMR, MR^4.0^, and MR^4.5^ (Table S7). Contrarily, higher eosinophil and basophil counts were associated with favorable achievement of MR^4.0^ and MR^4.5^. Based on the results of the multivariate analysis, male sex was an adverse prognostic factor for MMR, MR^4.0^, and MR^4.5^. Meanwhile, a higher baseline proportion of CD16⁺CD56⁺ NK cells was associated with favorable MMR outcomes. Regarding the achievement of MR^4.0^ and MR^4.5^, MMR achievement at 12 months and higher baseline NK-cell levels were identified as the predictive factors of MR^4.0^ and MR^4.5^ achievement. The EUTOS score predicted MR^4.0^ and showed a trend toward predicting MR^4.5^ (Table S8).

Altogether, 12 (25.0%) of the 48 patients experienced grade ≥3 AEs, including cardiovascular AEs (*n* = 6), infections (n = 3), and secondary malignancy (*n* = 1), while on dasatinib treatment (Table S9). Furthermore, 18 (37.5%) of the 48 patients were switched to a second-line therapy because of AEs or treatment resistance after a median of 36.0 months (IQR: 23.3–59.7 months). Only three patients switched treatment because of resistance (M244V mutation). Of the remaining patients, 13 switched treatment due to AEs and two switched for other reasons. Pleural effusion was the most common reason for switching from dasatinib treatment, with eight patients requiring a treatment change (grade 1, *n* = 2; grade 2, *n* = 6). Most cases of pleural effusion occurred in patients receiving relatively higher dasatinib doses (≥40 mg) (Table S10). Notably, no case of grade ≥3 pleural effusion was observed. Only events observed during dasatinib treatment are included.

**Fig. 2:**
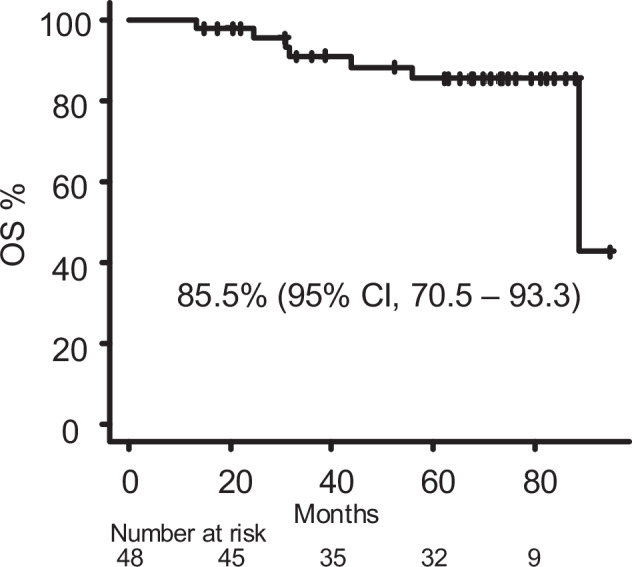
Overall survival (OS). Overall survival in the whole study population.

The 5-year OS rate was 85.5% (95% CI: 70.5–93.3%; Fig. 2). Among the seven deaths, only one was CML-related (development of BC), with most deaths caused by heart failure or other cancers (Table S11). Favorable OS was observed even among elderly patients with CML.

Finally, achieving treatment-free remission (TFR) is a key therapeutic goal in the management of patients with CML [8]. TFR was attempted in accordance with the 2020 European LeukemiaNet (ELN) recommendations, with the discontinuation criterion defined as a TKI therapy duration of >4 years for dasatinib and DMR duration (MR^4.0^) of >3 years in the general clinical setting [9]. Altogether, six of the 48 patients attempted TFR, whereas 11 met the eligibility criteria but continued the treatment with dasatinib. The patients’ median age was 78.5 years (range: 70–83 years), with an equal distribution of men and women (three each). All six patients were receiving dasatinib at a dose of 20 mg prior to treatment discontinuation. TFR was attempted at a median of 57.8 months (IQR: 49.3–63.9 months) from treatment initiation and 44.0 months (IQR: 41.0−47.9 months) from the MR^4.0^ achievement. During a median follow-up of 17.3 months (IQR: 13.5–18.8 months) after treatment discontinuation, all six patients maintained TFR without MMR loss (Fig. S2). Based on these findings, TFR can be successfully achieved even in older patients if the ELN criteria for TFR are fulfilled [2]. Given that all patients achieving TFR had been treated with low-dose dasatinib, patients with high TKI sensitivity may be more likely to successfully achieve TFR [10].

Our analysis showed that treatment with low-dose dasatinib could maintain durable efficacy and safety over the long term. The achievements of MMR, MR^4.0^, and MR^4.5^ were consistent with the 5-year outcomes of pivotal frontline trials [4]. Notably, low-dose dasatinib treatment provided sustained therapeutic benefits even among the elderly patients.

Based on our analysis, the achievement of MMR at 12 months was significantly associated with a higher probability of subsequently attaining DMR. This finding is consistent with the results of the CML Study IV, reporting that patients achieving MMR at earlier time points had a higher probability of subsequently attaining DMR [11]. Our study findings support the clinical relevance of the 12-month MMR milestone for identifying patients who are likely to achieve DMR, a prerequisite for TFR. NK cell-mediated immunity [12] or sex-specific immune differences [13] may influence responses to TKI therapy, and basophils might act as immunomodulatory effector cells [14], as also demonstrated in the present cohort.

Regarding safety, the proportion of grade 3–4 AEs increased from 23.0% at 12 months to 29.2% during the extended follow-up. Of note, the incidence of newly developed grade 3–4 AEs after the initial report was low. Notably, eight patients required a switch to second-line treatment due to pleural effusion. Dasatinib-associated pleural effusion is not limited to the early treatment phase and may develop at any time during therapy, resulting in an increased cumulative incidence with long-term follow-up [15]. Importantly, pleural effusion primarily occurred in patients receiving dasatinib at a dose of ≥40 mg, whereas only two patients developed pleural effusion while on 20 mg dasatinib. Therefore, a low-dose treatment of dasatinib may be associated with a lower incidence of pleural effusion, although a definitive conclusion cannot be drawn from our study due to its limited sample size.

In conclusion, the present study showed the long-term efficacy and safety of low-dose dasatinib. A dose-adapted dose of dasatinib can be an appropriate and clinically reasonable option in elderly patients with CML.

## Supplementary information

Supplemental material
