# Supplementary material for: Long-term outcomes of low-dose dasatinib in older patients with chronic myeloid leukemia in chronic phase: an extended follow-up of the DAVLEC phase 2 trial
Source: Blood Cancer J. 2026 May 28;16(1):83. doi: 10.1038/s41408-026-01526-7 (PMC13219402; doi:10.1038/s41408-026-01526-7)
Supplement: Supplementary file 1 — Supplemental material [file 41408_2026_1526_MOESM1_ESM.pdf]

Supplementary Material

Supplementary Methods

T- and natural killer (NK)-cell profiles were analyzed at baseline and at 12 months after treatment initiation at a single central laboratory (Bio Medical Laboratories, Tokyo, Japan). Complete blood counts and differential leukocyte counts were measured using an automated hematology analyzer. The lymphocyte fraction was determined via flow cytometry based on forward scatter versus side scatter gating. Subsequently, to assess T- and NK-cell profiles, a two- or three-color flow cytometry was performed using the FACSCalibur cytometer and BD CellQuest software (version 3.3; BD Biosciences, Franklin Lakes, NJ, the USA). The T- and NK-cell subsets were defined as follows: CD4 T cells ($CD4^{+}CD8^{-}$), CD8 T cells ($CD3^{+}CD8^{+}$), NK cells ($CD3^{-}CD56^{+}$ and $CD16^{+}CD56^{+}$), T-cell large granular lymphocytes ($CD3^{+}CD57^{+}$), NK-cell large granular lymphocytes ($CD56^{+}CD57^{+}$), and regulatory T cells (Tregs, $CD4^{+}CD25^{+}CD127^{low}$).

Table S1. Patient characteristics

	Median	IQR, %
Age at diagnosis	78	74.8-83.5
Sex		
male	32	66.7%
female	16	33.3%
Height, cm	160	153 – 163
Body weight, kg	55.5	50.4 – 63.3
ECOG performance status		
0	39	81.3%
1	8	16.7%
2	1	2.1%
BCR::ABL1 mRNA IS %	103.3	89.2-125.2%
Sokal		
Low	2	4.4%
Intermediate	35	77.8%
High	8	17.8%
Hasford		
Low	12	26.7%
Intermediate	32	71.1%
High	1	2.2%
EUTOS		
Low	41	87.2%
High	6	12.8%
ELTS		
Low	5	10.6%
Intermediate	25	53.2%
High	17	36.2%
Splenomegaly	7	14.6%
Comorbidity	31	64.6%
Cardiovascular disease risk	26	54.2%
Cardiovascular disease	12	25.0%
Other cancer	17	35.4%

IQR, interquartile range; IS, international scale

Table S2. Patient comorbidities

		n	(%)
Patients with cardiovascuar disease (CVD)		12	25.0
	Acute coronary disease	5	10.4
	Atrial fibrillation	4	8.3
	Chronic heart failure	2	4.2
	Cerebral infarction	2	4.2
	Arteriosclerosis obliterans	1	2.1
	Atrioventricular block	1	2.1
	Abdominal aortic aneurysm	1	2.1
Patients with CVD risk factors		26	54.2
	Hypertension	18	37.5
	Hyperlipidemia	12	25.0
	Diabetes mellitus	12	25.0
Chronic obstructive pulmonary disease		2	4.2
Asthma		1	2.1
Patients with other malignancies		17	35.4
	Colon cancer	6	12.5
	Prostate cancer	5	10.4
	Gastric cancer	4	8.3
	Esophageal cancer	2	4.2
	Thyroid cancer	2	4.2
	Breast cancer	1	2.1
	Hypopharyngeal cancer	1	2.1

Some patients had overlapping CVDs or cancer types.

Table S3. Dasatinib dose at MMR, $MR^{4.0}$ and $MR^{4.5}$

Dose at MMR (mg)	n = 40
10	1
20	25
40	9
50	4
60	1
70	1
Dose at $MR^{4.0}$ (mg)	n = 30
20	21
40	6
50	1
60	1
70	1
Dose at $MR^{4.5}$ (mg)	n = 24
10	1
20	18
40	3
60	1
70	1

MMR, major molecular response; $MR^{4.0}$, molecular response with a 4.0-log reduction; $MR^{4.5}$, molecular response with a 4.5-log reduction

Table S4. T cells and NK in peripheral blood at 0-month and 12-month since dasatinib initiation

	0M median, %	IQR	12M median, %	IQR	P value 0M vs 12M
CD4+CD8-	18.2	15.0 – 22.5	28.6	19.4 – 31.9	P < 0.001
CD3+CD8+	8.6	5.3 – 13.0	14.5	8.8 – 22.8	P < 0.001
CD3-CD56+	13.6	9.4 – 16.7	34.4	23.7 – 40.2	P < 0.001
CD16+CD56+	11.5	8.0 – 14.9	26.1	19.6 – 36.5	P < 0.001
CD3+CD57+	8.5	4.3 – 12.2	14.7	7.4 – 20.3	P < 0.001
CD56+CD57+	10.3	7.8– 13.7	25.1	18.7 – 36.0	P < 0.001
Treg (in CD4+)	5.8	3.7 – 7.2	5.1	4.0 – 7.0	P = 0.725

M, month; IQR, interquartile range; Treg, regulatory T cell; student t-test

Table S5. Univariate analyses of immune cell factors affecting MMR, $MR^{4.0}$ and $MR^{4.5}$ at baseline

	MMR			$MR^{4.0}$			$MR^{4.5}$		
	HR	95% CI	P value	HR	95% CI	P value	HR	95% CI	P value
CD4+CD8- ≥ 18.2%	1.316	0.702 – 2.466	0.392	1.322	0.642 – 2.723	0.449	1.204	0.537 – 2.697	0.652
CD3+CD8+ ≥ 8.6%	0.579	0.308 – 1.089	0.090	0.763	0.372 –1.567	0.462	0.805	0.351 – 1.845	0.608
CD3-CD56+ ≥ 13.6%	1.956	1.024 – 3.736	0.042	1.360	0.662 – 2.794	0.403	1.259	0.562 – 2.818	0.576
CD16+CD56+ ≥ 11.5%	2.007	1.059 – 3.802	0.033	1.227	0.598 – 2.517	0.577	1.054	0.471 – 2.359	0.899
CD3+CD57+ ≥ 8.5%	1.198	0.631 – 2.274	0.581	0.671	0.327 – 1.379	0.278	1.124	0.472 – 2.678	0.791
CD56+CD57+ ≥ 10.3%	1.753	0.918 – 3.347	0.089	1.058	0.512 – 2.186	0.878	0.849	0.370 – 1.949	0.699
Treg%(CD4+) ≥ 5.81	0.827	0.444– 1.542	0.550	0.710	0.346 – 1.459	0.352	0.831	0.372 – 1.856	0.651

MMR, major molecular response; $MR^{4.0}$, molecular response with a 4.0-log reduction; $MR^{4.5}$, molecular response with a 4.5-log reduction; HR, hazard ratio; 95% CI, 95% confidence interval. Cox proportional hazards models. Continuous variables were dichotomised at their median values.

Table S6. Univariate analyses of immune cell factors affecting MMR, $MR^{4.0}$ and $MR^{4.5}$ at 12 months after dasatinib initiation

	MMR			$MR^{4.0}$			$MR^{4.5}$		
	HR	95% CI	P value	HR	95% CI	P value	HR	95% CI	P value
CD4+CD8- ≥ 28.6%	1.095	0.571 – 2.099	0.785	0.881	0.413 – 1.879	0.744	0.907	0.384 – 2.142	0.823
CD3+CD8+ ≥ 14.5%	0.998	0.522 – 1.908	0.994	1.171	0.547 – 2.506	0.684	1.465	0.605 – 3.545	0.398
CD3-CD56+≥ 34.4%	1.357	0.710 – 2.597	0.356	1.112	0.521 – 2.369	0.784	0.899	0.380 – 2.125	0.808
CD16+CD56+ ≥ 26.1%	1.357	0.710 – 2.597	0.356	1.112	0.521 – 2.369	0.784	0.899	0.380 – 2.125	0.808
CD3+CD57+ ≥ 14.7%	0.891	0.466 – 1.702	0.727	0.755	0.355 – 1.609	0.467	1.124	0.472 – 2.678	0.791
CD56+CD57+ ≥ 25.1%	1.168	0.612 – 2.230	0.638	1.284	0.600 – 2.749	0.520	1.229	0.516 – 2.926	0.641
Treg%(CD4+) ≥ 5.1%	0.715	0.374 – 1.366	0.310	0.721	0.338 – 1.538	0.398	0.640	0.269 – 1.525	0.314

MMR, major molecular response; $MR^{4.0}$, molecular response with a 4.0-log reduction; $MR^{4.5}$, molecular response with a 4.5-log reduction; HR, hazard ratio; 95% CI, 95% confidence interval. Cox proportional hazards models. Continuous variables were dichotomised at their median values.

Table S7. Univariate analysis of factors affecting MMR, $MR^{4.0}$ and $MR^{4.5}$

	MMR			$MR^{4.0}$			$MR^{4.5}$		
	HR	95% CI	P value	HR	95% CI	P value	HR	95% CI	P value
WBC, ≥ 20.9×10^9/L	0.629	0.306 – 1.295	0.208	0.737	0.327 – 1.660	0.461	0.675	0.274 – 1.664	0.393
Hb ≥ 125 g/L	1.094	0.933 – 1.283	0.271	1.073	0.892– 1.291	0.456	1.106	0.891 – 1.373	0.363
PLT ≥ 510×10^9/L	1.663	0.895 – 3.088	0.108	2.047	0.978– 4.284	0.057	2.169	0.917 – 5.134	0.078
Blast positive	0.800	0.398– 1.609	0.531	0.859	0.382 – 1.928	0.712	0.551	0.213 – 1.427	0.220
Baso ≥ 6.5%	1.129	0.605 – 2.107	0.702	2.774	1.290 – 5.967	0.009	3.144	1.300 – 7.604	0.011
Eo ≥ 2.5%	1.849	0.985 – 3.47	0.056	3.396	1.552 – 7.433	0.002	2.899	1.191– 7.057	0.019
Splenomegaly positive	0.707	0.275 – 1.819	0.473	1.092	0.372 – 3.207	0.872	0.875	0.260 – 2.949	0.830
Age ≥ 78	0.850	0.457– 1.578	0.606	1.031	0.499 – 2.131	0.933	0.977	0.436 – 2.186	0.954
Sex, male	0.417	0.218 – 0.799	0.008	0.394	0.191 – 0.815	0.012	0.2718	0.118 – 0.624	0.002
Hasford, low	1.337	0.660 – 2.694	0.422	1.035	0.37 – 2.451	0.937	1.113	0.434 – 2.853	0.823
EUTOS, low	1.558	0.554 – 4.386	0.401	0.994	0.345 – 2.861	0.991	0.928	0.274 – 3.145	0.904

MMR, major molecular response; $MR^{4.0}$, molecular response with a 4.0-log reduction; $MR^{4.5}$, molecular response with a 4.5-log reduction; HR, hazard ratio; 95% CI, 95% confidence interval; WBC, white blood cell; Hb, hemoglobin; PLT, platelet; Baso, basophil, Eo, eosinophil. Cox proportional hazards models. Continuous variables were dichotomised at their median values.

Table S8. Multivariate analysis of factors associated with molecular responses

Multivariate analysis for MMR	HR	95% CI	p value
Male sex	0.257	1.328 – 5.000	0.005
CD16+CD56+NK at 0 months ≥ 11.5%	2.120	1.103 – 4.075	0.002
Multivariate analysis for $MR^{4.0}$	HR	95% CI	p value
Optimal response at 12 months	8.801	2.971 – 26.070	<0.001
Basophil ≥ 6.5%	5.817	2.239 – 15.110	<0.001
CD3-CD56+NK at 0 months ≥13.6%	3.615	1.447 – 9.033	0.006
EUTOS low risk	5.295	1.285 – 21.820	0.021
Male sex	0.387	0.169 – 0.889	0.025
Multivariate analysis for $MR^{4.5}$	HR	95% CI	p value
Optimal response at 12 months	5.954	1.681 – 21.090	0.006
Basophil ≥ 6.5%	5.386	1.780 – 16.300	0.003
CD3-CD56+NK at 0 months ≥ 13.6%	2.979	1.125 – 7.890	0.029
EUTOS low risk	3.917	0.899 – 17.060	0.069
Male sex	0.245	0.094 – 0.638	0.004

MMR, major molecular response; HR, hazard ratio; 95% CI, 95% confidence interval; NK, natural killer cell; Cox proportional hazards models. Continuous variables were dichotomised at their median values.

Table S9. Grade ≥3 adverse events and dasatinib dose

Patient ID	Adverse Events	Dasatinib dose (mg)
DAV005	Lower gastrointestinal bleeding and right pontine infarction	40
DAV011	QT prolongation	20
DAV012	Headache	40
DAV014	Diarrhea	20
DAV017	Atherothrombotic cerebral infarction	20
DAV019	Heart failure	On nilotinib
DAV022	Acute cholecystitis	40
DAV024	Pneumonia	20
DAV027	Lung cancer	On bosutinib
DAV028	Bilateral lower extremity arteriosclerosis obliterans	60
DAV032	Pneumonia	20
DAV037	Heart failure	20
DAV040	B-cell lymphoma	40
DAV043	Intracranial hemorrhage	40

Table S10. Reasons for switching from dasatinib and dasatinib dose

Patient ID	Reasons
DAV002	Pleural effusion (grade 2, 40 mg)
DAV007	Economic reason (40 mg)
DAV009	Pruritus (grade 2, 20 mg)
DAV011	QT prolongation (grade 3, 20 mg)
DAV013	Pleural effusion (grade 1, 50 mg)
DAV015	Pleural effusion (grade 2, 40 mg)
DAV019	Resistant (M244V, 20 mg)
DAV024	Pleural effusion (grade 2, 20 mg)
DAV027	Resistant (40 mg)
DAV028	Concomitant acid-suppressive therapy (60 mg)
DAV029	Pleural effusion (grade 1, 20 mg)
DAV035	Suspicious for pulmonary hypertension (20 mg)
DAV037	Heart failure (grade 3, 20 mg)
DAV040	B-cell lymphoma (grade 3, 40 mg)
DAV050	Pleural effusion (grade 2, 40 mg)
DAV052	Resistant (50 mg)
DAV055	Pleural effusion (grade 2, 40 mg)
DAV056	Pleural effusion (grade 2, 50 mg)

Table S11. Cause of death

Patient ID	Cause of death
DAV002	Multiple organ failure
DAV003	Gastric cancer
DAV019	Heart failure
DAV022	Acute cholangitis
DAV026	Heart failure
DAV027	Lung cancer
DAV037	CML-blast crisis

CML, chronic myeloid leukemia

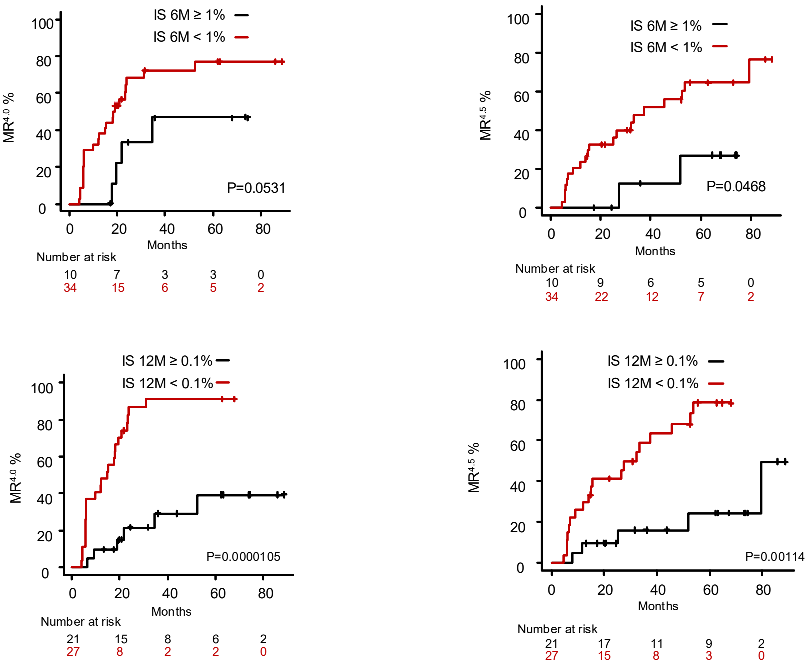

Figure S1. Cumulative incidence of $MR^{4.0}$ and $MR^{4.5}$ according to *BCR::ABL1* mRNA International Scale (IS) at 6 and 12 months.

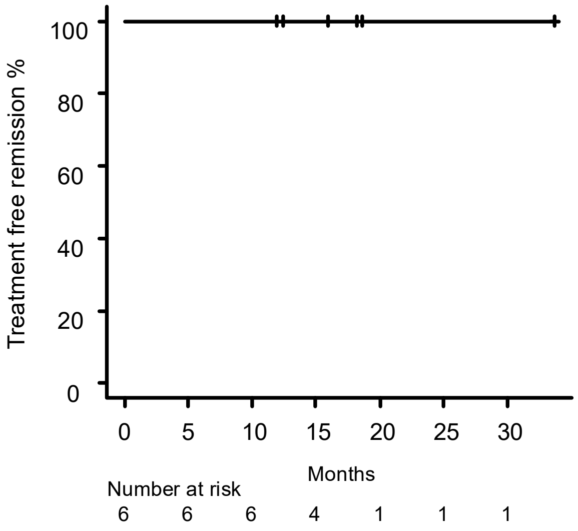

Figure S2. Treatment-free remission after discontinuation of dasatinib
